# PLK1 Mitigates Intervertebral Disc Degeneration by Delaying Senescence of Nucleus Pulposus Cells

**DOI:** 10.3389/fcell.2022.819262

**Published:** 2022-03-14

**Authors:** Zhenlei Zhang, Yizhen Huang, Nizhen Xu, Jianle Wang, Teng Yao, Yining Xu, Di Qiao, Jun Gao, Shuying Shen, Jianjun Ma

**Affiliations:** ^1^ Department of Orthopaedic Surgery, Sir Run Run Shaw Hospital, Zhejiang University School of Medicine, Hangzhou, China; ^2^ Key Laboratory of Musculoskeletal System Degeneration and Regeneration Translational Research of Zhejiang University Zhejiang Province, Hangzhou, China; ^3^ Department of Head and Neck Surgery, Institute of Micro-Invasive Surgery of Zhejiang University, Sir Run Run Shaw Hospital, Medical School, Hangzhou, China; ^4^ Shaoxing University School of Medicine, Shaoxing, China

**Keywords:** plk1, intervertebral disk degeneration, p53, cell senescence, cell proliferation

## Abstract

Intervertebral disc degeneration (IVDD) is the primary cause of low back pain; however, the molecular mechanisms involved in the pathogenesis of IVDD are not fully understood. Polo-like kinase 1 (PLK1) plays numerous roles in the cell cycle, including in cell proliferation and senescence. To investigate the involvement of PLK1 in IVDD, we used patient tissues and an animal model of IVDD. Samples were analyzed via immunoblotting, quantitative real-time polymerase chain reaction (qPCR), immunofluorescence, and immunohistochemistry. Our results demonstrated that PLK1 expression was decreased in nucleus pulposus cells (NPCs) of degenerative IVDs. The inhibition of PLK1 kinase activity in normal NPCs increased the expression of p53 protein, inhibited cell proliferation, and induced senescence. Our results suggest that PLK1 regulates the degeneration of the IVD through p53, revealing the function and mechanism of PLK1 in IVDD and providing a theoretical basis and experimental evidence for the potential treatment of low back pain.

## Introduction

Low back pain (LBP) is one of major disabilities worldwide, currently affecting approximately 632 million people (Andersson, 1999; Vos et al., 2012). It is very likely that due to the changes in population and lifestyle, the social and economic burden of LBP will continue to escalate (Dagenais et al., 2008). Even though there are numerous triggers for LBP, several studies have confirmed that intervertebral disc degeneration (IVDD) is the main underlying cause (Chou et al., 2011; Takatalo et al., 2011).

IVD is a multi-tissue organ that consists of bone endplates, cartilage endplates, central nucleus pulposus (NP), and annulus fibrous (Pattappa et al., 2012). It has been established that the pathogenesis of IVDD is a very complex process initiated by several factors, including aging, genetic factors, and cell apoptosis (Vo et al., 2010). Type II collagen and aggrecan are degraded by matrix metalloproteinases (MMPs), disintegrins, and metalloproteinases with thrombospondin motifs (ADAMTS) and have been shown to play a major role in joint inflammation. It has been reported that in degenerative disc tissue samples, the levels of type II collagen and proteoglycan (PG) are reduced (Lyons et al., 1981), whereas the expression levels of MMPs and second family of metalloproteinases (ADAMs), such as ADAMTS4 and 5 (Liu et al., 1991; Roughley, 2004) are increased. Furthermore, the synthesis of extracellular matrix (ECM) (PG and type II collagen) is markedly decreased, and the hydration of the intervertebral disc ECM is reduced, further aggravating the degeneration of NP cells (NPCs) (Hoyland et al., 2008).

NPCs synthesize and secrete ECM and, therefore, are the cells that maintain normal physiological functions of the IVD (Freemont, 2009; Millward-Sadler et al., 2009). The decreased numbers and dysfunction of NPCs are directly responsible for the development and progression of IVDD and, in turn, are correlated with LBP severity (Cheung et al., 2009; Feng et al., 2016). Therefore, to develop effective treatments for LBP, it is necessary to elucidate the molecular mechanisms underlying the pathogenesis of IVDD.

The polo gene, encoding Polo kinase, was discovered in Drosophila in 1988 (Sunkel and Glover, 1988). Currently, polo-like kinase 1 (PLK1), a protooncogene, is one of the anti-cancer drug targets and has been extensively studied due to its role in cancer development (Lee et al., 2015). The N-terminus of PLK1 contains a serine/threonine kinase domain, and the C-terminus contains a polo-box domain (Strebhardt, 2010). PLK1 plays a regulatory role in several stages of the eukaryotic cell cycle (Glover et al., 1998), including mitosis and cytokinesis (Luo and Liu, 2012).

We hypothesized that PLK1 is involved in the proliferation of NPCs and, therefore, in the pathogenesis of IVDD. In this study, we tested this hypothesis and investigated the molecular mechanism of PLK1 function in IVDD using patient tissues and an animal model of IVDD.

## Results

### PLK1 Expression is Decreased in NPCs in IVDD

**FIGURE 1:**
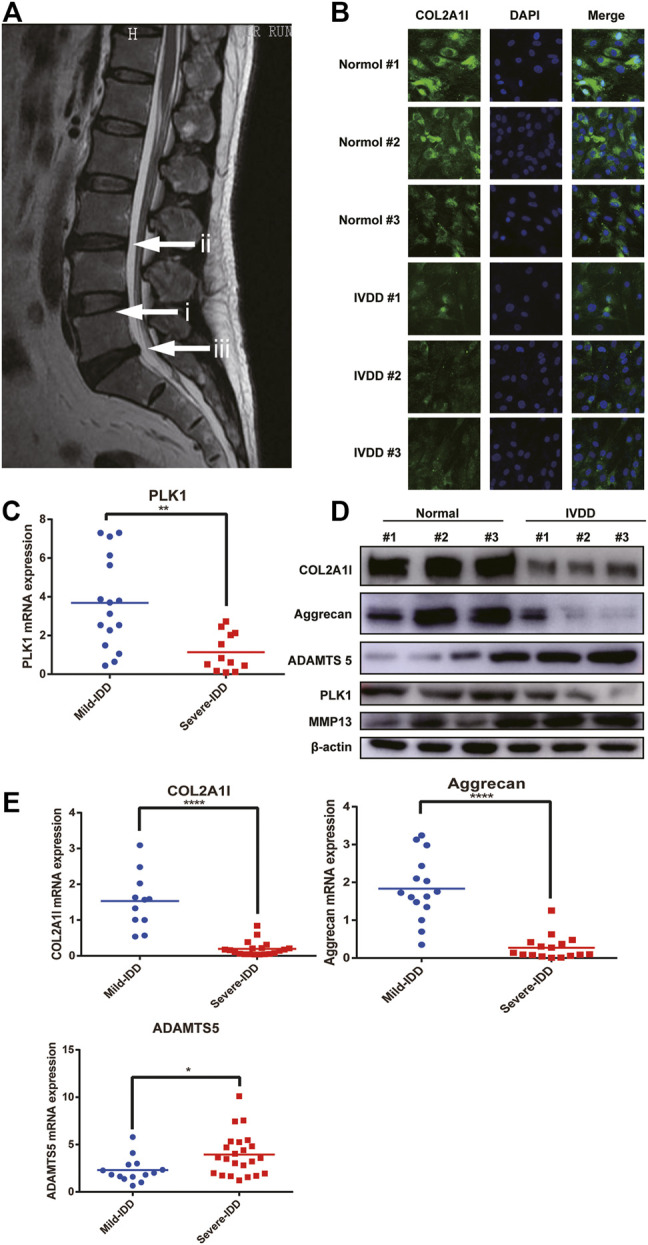
PLK1 expression is decreased in NPCs in IVDD. **(A)** Preoperative MRI scan of the spine of IVDD. (i) refers to normal intervertebral disc tissue, and (ii, iii) refers to degenerated intervertebral disc tissue. **(B)** The expression of collagen in NPCs from normal and degenerate IVDs. **(C)** The mRNA expression of PLK1 in NPCs isolated from different degenerated IVDs. **(D)** The protein expression of collagen, aggrecan, ADAMTS5, PLK1, and MMP13 in NPCs isolated from different degenerated IVDs. **(E)** QPCR analyses were performed for collagen, aggrecan, and ADAMTS5. Data are shown as the mean ± SD, *p < 0.05, **p < 0.01, **p < 0.001, ****p < 0.0001. SD, standard deviation.

To investigate the molecular mechanisms involved in the pathogenesis of IVDD, disc specimens were collected from patients undergoing fracture surgeries and disc degeneration surgeries. Representative magnetic resonance imaging (MRI) scans of patients with IVDD are shown in Figure 1A. The results of immunofluorescence experiments demonstrated that the expression of collagen was reduced (Figure 1B). We have examined their growth rates and senescence in normal and degenerative NP cells, as shown in Supplementary Figures S1A,B.

It has been previously reported that IVDD leads to decreased cell proliferation. Since PLK1 plays a major role in cell proliferation, we hypothesized that PLK1 was involved in the pathogenesis of IVDD. Quantitative real-time polymerase chain reaction (qPCR) and western blotting results showed that, compared with that in normal NPCs, the expression of PLK1 in NPCs isolated from the degenerated IVDs was significantly reduced (Figures 1C,D). In patients with IVDD, the levels of collagen II and aggrecan were decreased, whereas the expression levels of ADAMTS5 and MMP3 were increased (Figures 1D,E). These results indicated that PLK1 was involved in IVDD.

### PLK1 Knockdown Inhibits Proliferation and Induces Senescence in NPCs, Whereas PLK1 Overexpression Reverses These Effects

To investigate the role of PLK1, we performed RNA interference experiments in NPCs (Supplementary Figure S2A). Our results showed that the knockdown of PLK1 significantly reduced the protein and gene of collagen II and aggrecan and significantly increased the expression of ADAMTS5, MMP3, and MMP13 (Supplementary Figures S2B,C).

**FIGURE 2:**
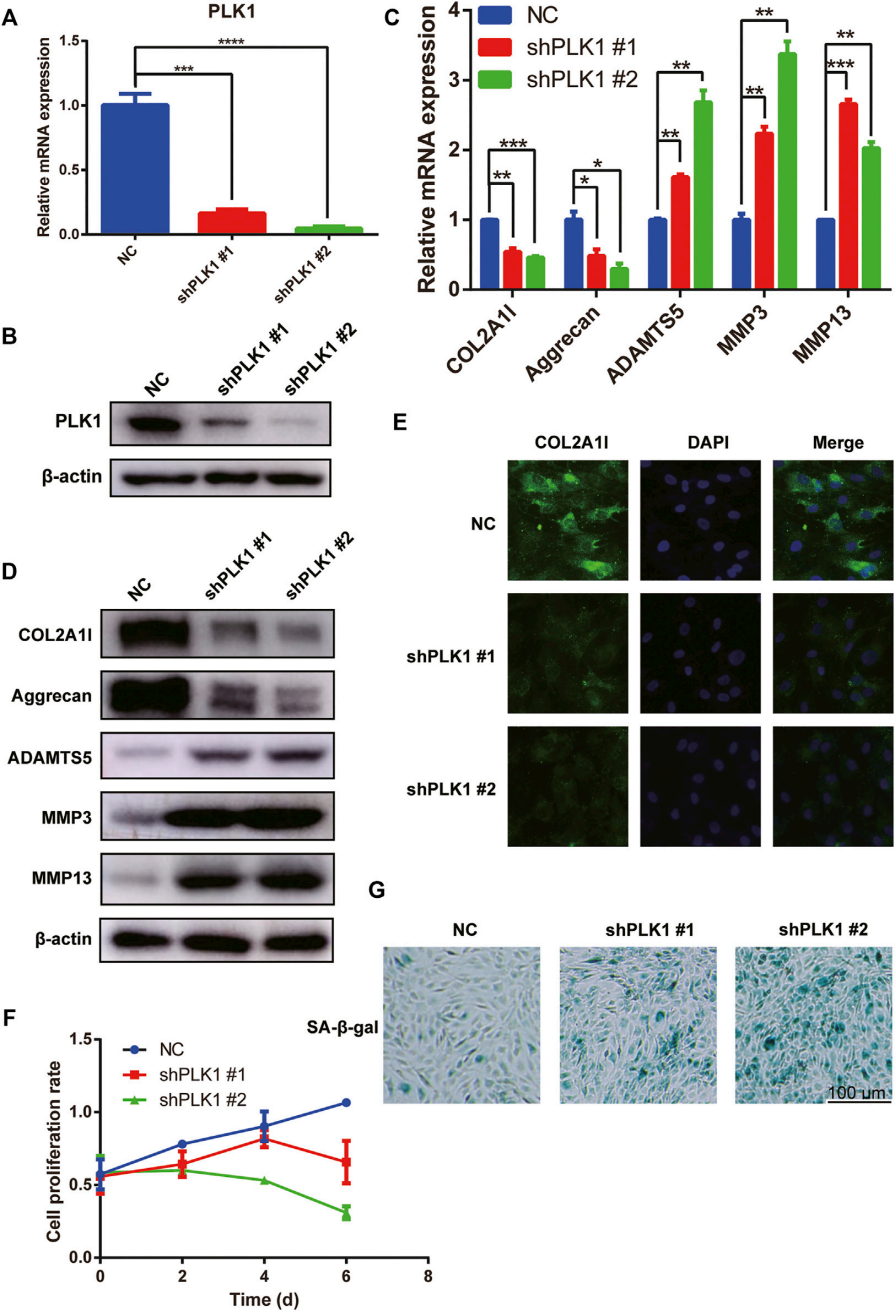
PLK1 knockdown inhibits proliferation and induces senescence in NPCs. **(A,B)** The efficiency of PLK1 shRNAs. **(C,D)** The mRNA and protein expression of collagen, aggrecan, ADAMTS5, MMP3, and MMP13 after knocking down PLK1 in NPCs. **(E)** The collagen was detected by immunofluorescence, and blue fluorescence represents the nucleus, and green fluorescence represents collagen. **(F)** CCK8 assay was used to detect cell proliferation at the 0, 2nd, 4th, and 6th day after steadily knocking down PLK1 using shRNA. **(G)** Senescence of NPCs was measured by SA-β-Gal assay after knockdown by PLK1 shRNA in NPCs. All experiments were performed in duplicates, and data are reported as the mean ± SD. *p < 0.05, **p < 0.01, ***p < 0.001, ****p < 0.0001. SD, standard deviation.

Next, we evaluated the effect of PLK1 on the proliferation of NPCs. For this purpose, we used PLK1 shRNA to continuously downregulate the expression of PLK1 (Figures 2A,B). qPCR, western blotting, and immunofluorescence results showed that the levels of collagen II and aggrecan were significantly decreased after PLK1 was knocked down, whereas the levels of ADAMTS5, MMP3, and MMP13 were significantly increased (Figures 2C–E). Cell Counting Kit 8 (CCK8) assays demonstrated that the growth rate of NPCs after PLK1 knockdown was reduced compared to that in the control group, and this inhibitory effect was even more pronounced at longer time points (Figure 2F). These results indicated that the knockdown of PLK1 decreased the proliferation of NPCs.

As cell proliferation in the knockdown group was significantly reduced, we evaluated cell senescence using SA-β-gal staining. Our results showed that PLK1 gene knockdown increased NPC senescence (Figure 2G). Therefore, these findings indicated that reduced PLK1 levels inhibited proliferation and induced the senescence of NPCs.

**FIGURE 3:**
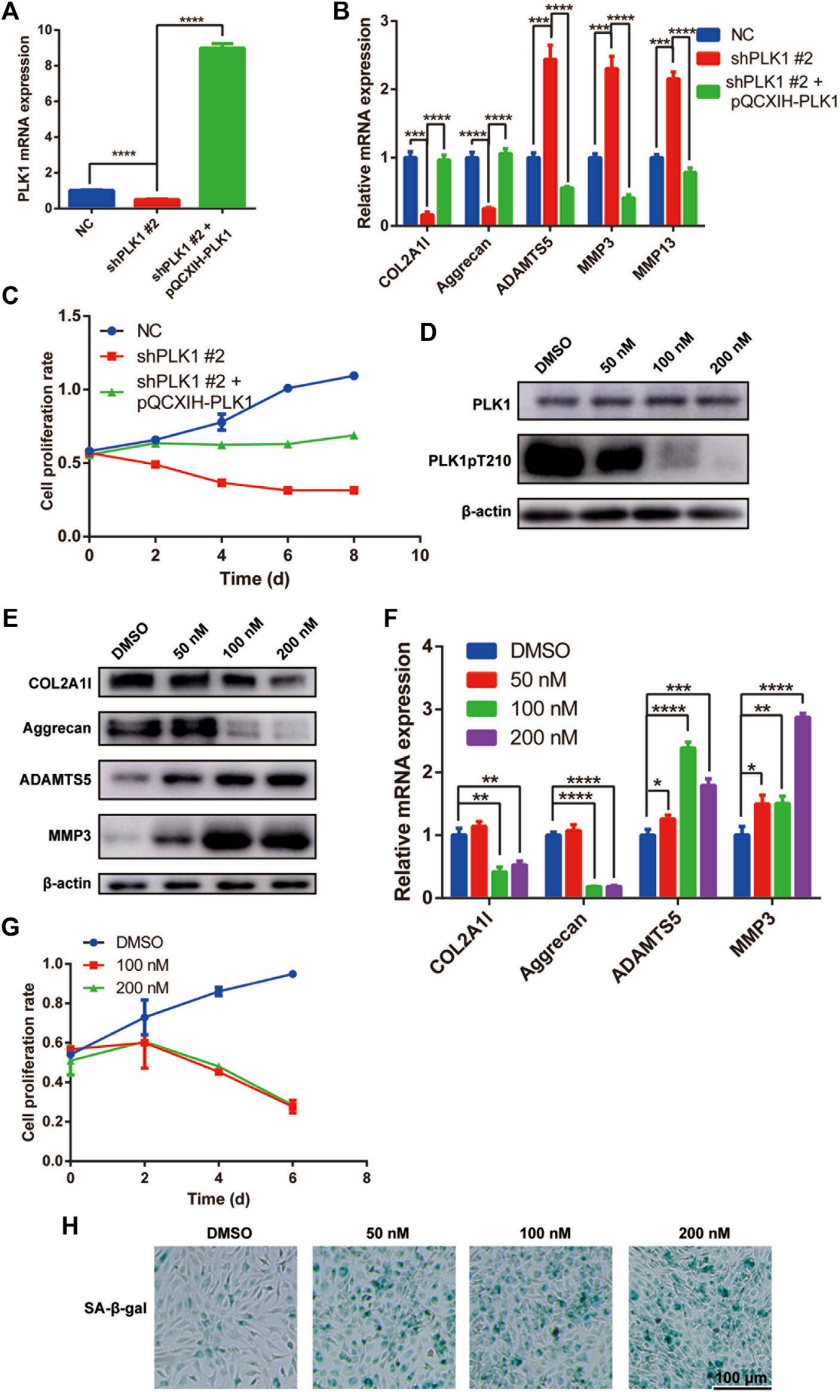
PLK1 overexpression reverses the effects caused by PLK1 shRNAs, and the PLK1 kinase inhibitor volasertib inhibits proliferation and induces senescence in NPCs. **(A)** The efficiency of PLK1 knockdown (shPLK1 #2) and overexpression (pQCXIH-PLK1) in NPCs. **(B)** The mRNA expression of collagen, aggrecan, ADAMTS5, MMP3, and MMP13 in NPC transfected with shPLK1 #2 and pQCXIH-PLK1. **(C)** CCK8 assay was used to detect cell proliferation at the 0, 2nd, 4th, 6th, and 8th day after NPCs were transfected with PLK1 shRNA and overexpression virus. **(D)** The expression of PLK1 and PLK1 pT210 after inhibiting the PLK1 kinase activity. **(E,F)** The expression of collagen, aggrecan, ADAMTS5, and MMP3 after inhibiting the PLK1 kinase activity detected by qPCR and western blot. **(H)** CCK8 assay was used to detect cell proliferation at the 0, 2nd, 4th, and 6th day after treatment with 100 and 200 nM volasertib. **(H)** Senescence of NPCs was measured by SA-β-Gal assay after treatment with volasertib. All experiments were performed in duplicates, and data are reported as the mean ± SD. *p < 0.05, **p < 0.01, ***p < 0.001, ****p < 0.0001. SD, standard deviation.

To verify the role of PLK1 in NPC cells, we overexpressed PLK1 (pQCXIH-plk1) in the NPC cells transfected with shPLK1 #2 virus (Figure 3A). The overexpression of PLK1 rescued protein and gene expression levels of collagen, aggrecan, ADAMTS5, MMP3, and MMP13 (Figure 3B) and increased the cell proliferation rate (Figure 3C). These results confirmed that the defects of NPCs were indeed due to the decreased expression levels of PLK1.

### PLK1 Kinase Inhibitor Volasertib Inhibits the Proliferation and Induces Senescence of NPCs

To elucidate the role of PLK1 in NPCs, we used volasertib, a PLK1 inhibitor. First, to establish the optimal concentration that would inhibit PLK1 kinase activity, we performed dose response experiments in NPCs and detected the phosphorylation level of PLK1 T210, a marker of PLK1 kinase activity. Our results showed that at 100 and 200 nM, volasertib was most effective at inhibiting PLK1 kinase activity (Figure 3D). Furthermore, at these volasertib concentrations, gene and protein expression levels of collagen and aggrecan were significantly decreased, whereas the expression levels of ADAMTS5 and MMP3 were significantly increased (Figures 3E,F). In addition, NPC proliferation was inhibited in cells treated with volasertib (Figure 3G). Next, we evaluated cell senescence using SA-β-gal staining. Our results showed that the senescence of NPCs was increased in cells treated with the PLK1 kinase inhibitor (Figure 3H). Collectively, these results indicated that the kinase activity of PLK1 played an important role in NPC proliferation and ECM expression.

### PLK1 Inhibition Increases the Expression of p53 and p21

Our results demonstrated that the kinase activity of PLK1 is involved in IVDD. To investigate the underlying mechanisms, we used qPCR to evaluate the expression levels of other signaling proteins in NPCs isolated from normal and degenerated IVDs. We found that the expression of p53 in the degenerated NPCs was significantly increased (Supplementary Figure S3A).

As shown in Figure 3I, volasertib induced senescence in NPCs. It has been previously reported that there is a connection between cellular senescence and p53 expression. Therefore, we hypothesized that PLK1 could affect IVDD via p53-related signaling pathways.

**FIGURE 4:**
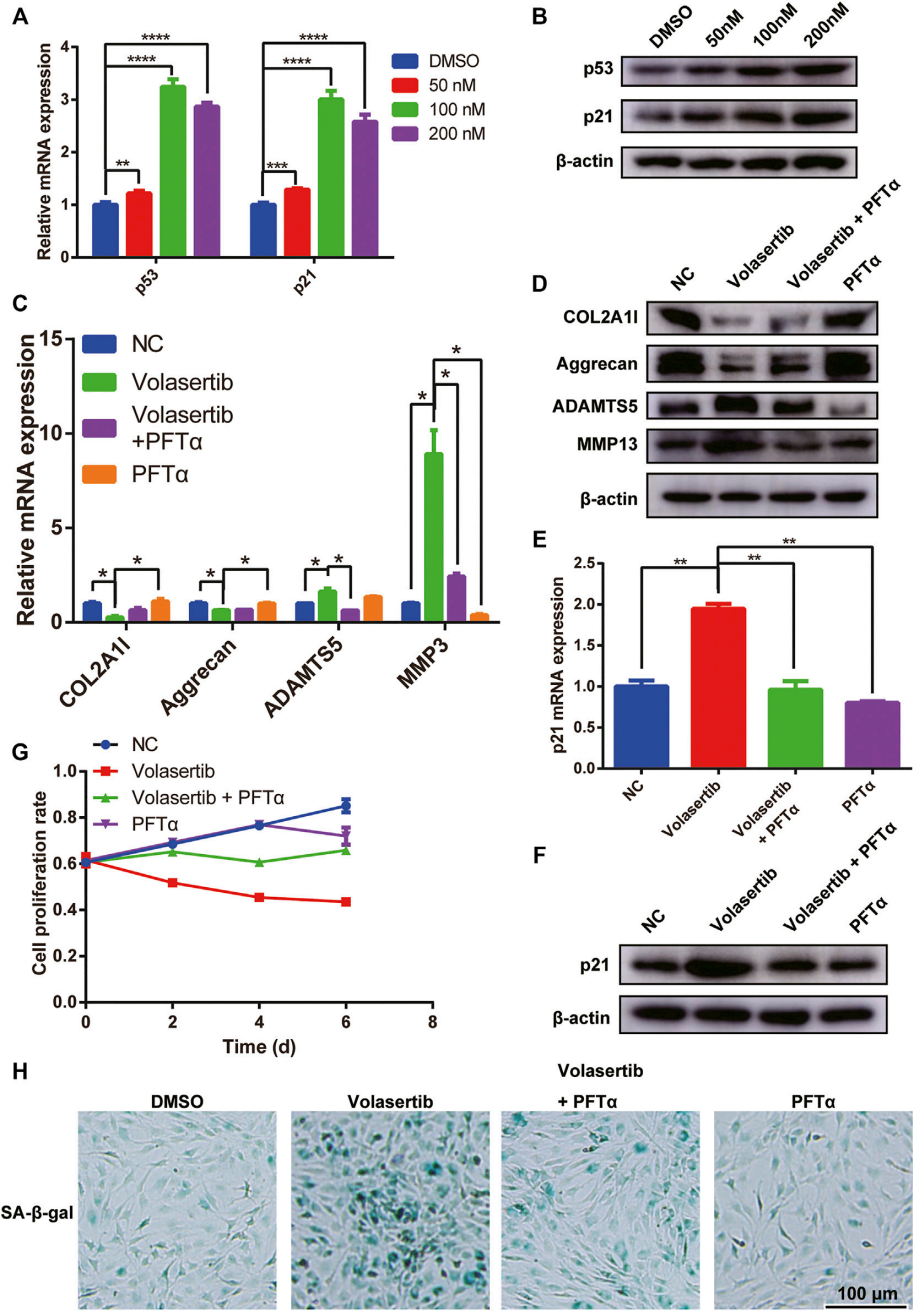
PLK1 inhibition increases the expression of p53 and p21. **(A,B)** The mRNA and protein expression of p53 and p21 after inhibiting the PLK1 kinase activity. **(C,D)** The mRNA and protein expression of collagen, aggrecan, ADAMTS5, MMP3 and MMP13 was detected in NPCs treated with volasertib or Pifithrin-α. **(E,F)** The expression of p21 was detected in NPCs treated with volasertib or Pifithrin-α. **(G)** CCK8 assay was used to detect cell proliferation at the 0, 2nd, 4th, and 6th day after treatment with volasertib or Pifithrin-α. **(H)** Senescence of NPCs was measured by SA-β-Gal assay after treatment with volasertib or Pifithrin-α. All experiments were performed in duplicates, and data are reported as the mean ± SD. *p < 0.05, **p < 0.01, ***p < 0.001, ****p < 0.0001. SD, standard deviation.

To test this hypothesis, we treated NPCs with volasertib and used qPCR and western blotting to detect p53 and p21. Our results showed that the gene and protein levels of both molecules were significantly increased (Figures 4A,B). The knockdown of PLK1 also upregulated the expression of p53 and p21 (Supplementary Figure S3B). To validate the specific role of PLK1 in IVDD, we used the p53 inhibitor Pifithrin-α (PFTα), which inhibits the function of p53. Our results showed that PFTα treatment rescued the levels of joint inflammation factors, such as collagen II, aggrecan, ADAMTS5, MMP3 and MMP13 (Figures 4C,D). Furthermore, the volasertib-mediated increase in p21 levels was reversed by PFTα treatment (Figures 4E,F). In addition, the proliferation rate of NPCs treated with volasertib was rescued by PFTα (Figure 4G). PFTα treatment also reversed the volasertib-induced senescence of NPCs (Figure 4H).

To elucidate the role of p53 in NPCs, we used p53 siRNA. We found that knockdown of p53 in NP cells from IVDD patients did not regenerate senescent cells into normal cells (Supplementary Figure S4C), but rescued normal NP cells that had become senescent due to the influence of volasertib (Supplementary Figures S4A,B). We believe that the senescence of NP cells in IVDD patients may be due to many factors, with the extent of phenotype alteration being very great, and p53 knockdown is not sufficient to rescue cells that have already undergone senescence.

Collectively, these results suggest that PLK1 affects IVDD through p53-related signaling pathways.

### 2.5 Inhibition of PLK1 Kinase Activity Aggravates IVDD in a Rat Model

**FIGURE 5:**
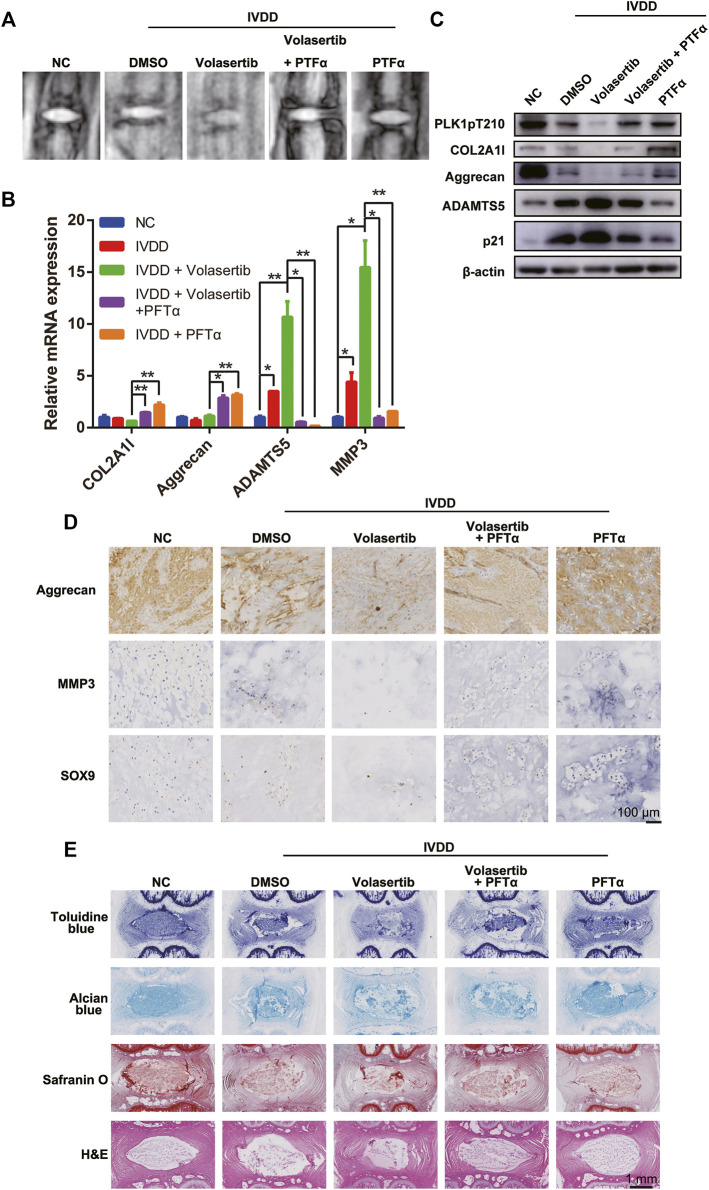
Inhibition of PLK1 kinase activity aggravates IVDD in a rat model. **(A)** MRI scans of rat tails. **(B)** The mRNA expression of collagen, aggrecan, ADAMTS5, and MMP3 was detected in rat tails treated with volasertib or Pifithrin-α. **(C)** The protein expression of PLK1 pT210, collagen, aggrecan, ADAMTS5 and p21 was detected in rat tail tissues. **(D)** Immunohistological staining of aggrecan, MMP3, and SOX9 in intervertebral disc from the different groups. **(E)** Toluidine blue, Alcian blue, safranin O, and H&E staining of intervertebral disc from the different groups. All experiments were performed in duplicates, and data are reported as the mean ± SD. *p < 0.05, **p < 0.01, ***p < 0.001, ****p < 0.0001. SD, standard deviation.

To investigate the role of PLK1 in IVDD *in vivo*, we used a rat needle puncture model. Five weeks after the surgery, the rats were treated with PFTα and volasertib via intravenous tail injection. Samples of the rat tail IVD were collected for imaging and histological analyses. Representative MRI scans of rat coccyges from sham and puncture groups are shown in Figure 5A.

Next, the rat NP samples were tested for the expression of arthritis-related and aging factors using qPCR, western blotting, and immunohistochemistry (IHC; Figures 5B–D). Our results showed that, compared with those in the normal NP, the expression levels of collagen, aggrecan, and SOX9 in the degenerated NP were decreased, whereas the expression levels of inflammatory factors (ADAMTS5 and MMP3) were increased. Furthermore, volasertib treatment exacerbated these effects; however, PFTα injection partially reversed these phenotypes.

Histological evaluation of rat IVD tissues demonstrated that, compared to that in the sham group, the number of NPCs in punctured group IVDs was reduced (Figure 5E). In summary, these results indicate that PLK1 plays a role in IVDD *in vivo* through the p53 signaling pathway.

## Discussion

In this study, we demonstrated that PLK1 plays an important role in the pathogenesis of IVDD in human tissues and in a rat needle puncture model. Our results showed that decreased expression levels of PLK1 induced IVDD via the p53-p21 signaling pathway. Furthermore, low PLK1 expression levels, in turn, resulted in increased expression levels of p53 and p21, leading to cell senescence, decreased proliferation, reduced collagen II production, increased ADAMTS5, MMP3 and MMP13 levels, and ultimately IVDD. Based on these results, we hypothesized that increased expression of PLK1 would induce cell proliferation, prevent cell senescence, and alleviate IVDD.

It has been reported that approximately 80% of adults are affected by LBP at some point in their lives (Deyo et al., 1990). Accurate pathophysiological diagnosis is the key to successful treatment. Numerous studies have shown that the main cause of LBP is IVDD (DePalma et al., 2011; Vergroesen et al., 2015), the condition caused by aging, genetic factors, NPC apoptosis, and other factors. For each individual, the influence of the environment on the degeneration is not clear; however, it is generally accepted that the accumulation of impacts and injuries, as well as psychosocial habits, plays a role in the pathogenesis of IVDD (Manchikanti et al., 2009).

The IVD, as a result of its unique ECM composition and structure, is a tissue with high water content (O'Halloran and Pandit, 2007; Li et al., 2008). Furthermore, due to its avascularity and low cell density (Pereira et al., 2013), the IVD is predisposed to degeneration. NP is the area with the highest water content in the IVD. During degeneration, the ECM of NPCs undergoes considerable changes depending on the status of these cells (Fontana et al., 2015). The loss of PGs, especially the loss of aggrecan, explains the inability of ECM to retain water for NPCs (Buckwalter, 1995; Roberts et al., 2006).

With age, the number of cells in the IVD decreases (Chen et al., 2006). NPCs are responsible for the synthesis and secretion of the ECM and, therefore, play important roles in normal physiological functions of IVD. Since the decrease of NPC number and function is the direct cause of IVDD, increasing the number of these cells and enhancing their function during aging could be a promising approach to treating IVDD.

PLK1 is a serine/threonine-protein kinase that plays a key role in the cell cycle and has different functions depending on the phase of the cell cycle. For example, during the prophase and prometaphase, PLK1 regulates spindle assembly; in the M phase, PLK1 removes cohesins, whereas in the telophase, it activates the anaphase-promoting complex/cyclosome (van de Weerdt and Medema, 2006). PLK1 is essential for the precise regulation of cell division and maintenance of mitosis, spindle assembly, and genome stability in response to DNA damage (Mundt et al., 1997; Lens et al., 2010).

In this study, we found that, compared with that in normal NP, the expression of PLK1 was significantly reduced in IVDD samples. Therefore, we hypothesized that PLK1 played a role in the development of this disease. Using siRNA and shRNA to knockdown PLK1 expression, we found that the proliferation of NPCs was inhibited, and cell senescence was induced, possibly accelerating IVDD. To determine whether this effect was due to the kinase activity of PLK1, we treated NPCs with volasertib, the inhibitor of PLK1 kinase activity. The inhibition of PLK1 reduced NPC proliferation and accelerated senescence. Next, to investigate the molecular mechanism of PLK1-induced IVDD, we evaluated several signaling proteins and demonstrated that p53 was significantly increased in NPCs treated with volasertib. We also found that the levels of the senescence-related protein p21 was increased; however, PFTα, a p53 inhibitor, reversed these effects. Therefore, these results indicated that PLK1 affected the proliferation and senescence of NPCs by regulating the expression levels of p53, thus further affecting the degeneration of the IVD.

To test our hypothesis *in vivo*, we used a needle puncture model to establish IVDD in rat tail vertebrae. The treatment of NPCs from rat tail vertebrae with volasertib and PFTα resulted in effects similar to those obtained *in vitro*. However, it is not clear whether PLK1 regulates the expression of p53 directly or indirectly. In cancer cells, the N-terminal kinase domain of PLK1 directly binds to the DNA-binding domain of p53, inhibiting the transactivation and pro-apoptotic function of p53 (Ando et al., 2004). In 2009, Yand et al. reported that PLK1 inhibits topoisomerase I binding protein (Topors)-mediated p53 SUMOylation via Topors phosphorylation, promoting p53 ubiquitination and accelerating p53 degradation (Yang et al., 2009). Since Topors was originally identified as a protein that binds to DNA topoisomerase I and p53 (Haluska Jr et al., 1999; Zhou et al., 1999), in our future experiments, we will investigate whether PLK1 could phosphorylate Topors in NPCs, leading to the inhibition of p53 expression and subsequent decrease of senescence and apoptosis, providing a theoretical basis for clinical treatment of IVDD.

In summary, using human IVDD samples and a rat model of IVDD, we showed that decreased expression of PLK1 induced IVDD. We also demonstrated that the reduced levels of PLK1 increased p53 expression, promoted NPC senescence, and induced IVD degeneration. These findings identify a possible therapeutic target for the treatment of LBP due to IVDD.

## Materials and Methods

### Ethical Approval

The animal experiments in this article are conducted in accordance with the “Guidelines for the Care and Use of Laboratory Animals”. These experiments have been approved by the Clinical Committee of Sir Run Run Shaw Hospital.

### Clinical Samples

The protocols used in collecting human nuleus pulposus tissues are provided by the Ethics Committee of Sir Run Run Shaw Hospital. We strictly follow the prescribed methods to obtain specimens. All patients signed the informed consent form.

The nucleus pulposus cells were extracted from patients’ resected specimens. Normal nucleus pulposus cells were from patients who had surgery due to scoliosis or thoracolumbar fracture, and the degenerated intervertebral disc cells were from patients who had degenerative disc disease.

### Human Tissues From Normal and Degenerated NP

The tissues of nucleus pulposus were washed several times with 0.9% normal saline until they are clean. The tissue specimens were cutted into small pieces of 0.5 mm in length in a sterile biological cabinet, then were incubated with type II collagenase solution (Invitrogen, Carlsbad, United States) at constant temperature incubator for 4–6 h. The tissues were centrifugated and washed with 0.9% normal saline and passed through a 10 μm strainer.

### Cell Culture

NP cells were cultured at a constant temperature and humidity incubator with 5% CO_2_ at 37°C and using Dulbecco’s modified Eagle’s medium (Gibco, Gaithersburg, United States) which was containing 10% Fetal Bovine Serum (FBS, Gibco, Gaithersburg, United States), 1% penicillin and streptomycin (Invitrogen, Carls-bad, United States). We treated NPCs with Pifithrin-α (10 μM) and different concentrations of Volasertib.

### Cell Viability Assay

We digested the nucleus pulposus cells with trypsin, seeded them on a 96-well plate with 8,000 cells in each well, and treated them with drugs after 24 h of culture.

Different concentrations of Volasertib or Pifithrin-α (10 μM) were used to the cells in 96-well plate. 1–8 days later, the nucleus pulposus cells were treated with 10 μl Cell Counting Kit-8 buffer (Beyotime, Shanghai, China) and 100 μl fresh medium. One hour later, we measured the absorbance at 450 nm wavelength (650 nm reference) with a Versamax microplate reader (Molecular Devices, Sunnyvale, CA, United States).

### Quantitative Real-Time Polymerase Chain Reaction

In this study, we use TRIzol reagent (Invitrogen, Carlsbad, CA, United States) to extract total RNA from human nucleus pulposus cells and rat nucleus pulposus tissues.

Total RNA was extracted from human nucleus pulposus cells or rat nucleus pulposus tissues by using the TRIzol RNA isolation protocol (Invitrogen, Inc., Carlsbad, CA, United States). AG Reverse Transcription Kit (Accurate biotechnology, Changsha, Hunan, China) was used to reverse transcribe total RNA into cDNA and SYBR Premix ExTaq II (Yeasen Biotechnology, Shanghai, China) SYBR was used for the reactions of qPCR. Then we used a ABI 7500 sequencing detection system (Applied Biosystems, Foster City, CA, United States) to measure the reactions and applied the β-actin as a control RNA expression level. Data were analyzed by GraphPad Prism (GraphPad Prism Software 6.0, United States). The primers we used in the experiments: β-actin, AGA​GCT​ACG​AGC​TGC​CTG​AC, PLK1, CAC​CAG​CAC​GTC​GTA​GGA​TT, MMP3, CCT​ACA​AGG​AGG​CAG​GCA​AG, MMP13, TCG​GCC​ACT​CCT​TAG​GTC​TT, COL2A1, CCA​GAT​GAC​CTT​CCT​ACG​CC, ADAMTS4, AAC​GTC​AAG​GCT​CCT​CTT​GG, ADAMTS5, CCG​GAG​CCA​CTG​CTT​CTA​TC, aggrecan, GGG​ACC​TGC​AAG​GAG​ACA​GAG, SOX9, GCT​CTG​GAG​ACT​TCT​GAA​CGA, p53, CCA​GGA​TGT​TGC​AGA​GTT​GTT​A, p21, TAT​TTT​GTC​CTT​GGG​CTG​CCT, p16, CTT​CGG​CTG​ACT​GGC​TGG.

### Western Blot Analysis

Total proteins of human NPCs or rat NP tissues were extracted with a lysis buffer containing 1% sodium dodecyl sulphate and 50 mM Tris-hydrochloride (HCl). The lysates of cell or tissues were boiled for 10 min in metal bath instrument and were quantified with Bicinchoninic Acid Protein Assay Kit (Sangon Biotec, Shanghai, China).

Different proteins in the samples can be separated by 6–12% sodium dodecyl sulphate polyacrylamide gel electrophoresis (SDS-PAGE), then all the proteins in the gel were transferred onto 0.22 μm polyvinylidene fluoride membranes (PVDF, Millipore, United States).

Five% skim milk was dissolved in a Tris-buffered saline (TBS) solution containing 0.1% Tween-20 (Invitrogen, San Diego, CA, United States), and blocked the membranes for 1 h. Then the membranes were incubated with suitable primary antibodies at 4°C overnight including anti-COL2A1l (sc-52658, Santa Cruz), anti-Aggrecan (13880-1-ap, Proteintech), anti-ADAMTS5 (ab41037, Abcam), anti-MMP3 (ab52915, Abcam), anti-MMP13 (ab39012, Abcam), anti-p53 (10442-1-AP, Proteintech), anti-p21 (10355-1-AP, Proteintech), anti-PLK1 (ab17057, Abcam), anti-PLK1 phospho T210 (ab155095, Abcam), anti-β-actin (66009-1-Ig, Proteintech).

Then the membranes were washed at least 4 times for 5 min per time. Afterwards, the membranes were incubated with the HRP-conjugated secondary antibodies for at least 1 h. At last, detecting all the bands with Enhanced ECL Chemiluminescent Substrate Kit (Yeasen, Shanghai, China).

### Agarose Nucleic Acid Gel Electrophoresis

Agarose nucleic acid gel electrophoresis was used to separate cDNA fragments of different sizes. The buffer contains Tris base, acetic acid and EDTA. The PowerPac Universal power (BIO-RAD, CA, United States) and Super DNA Marker (New England Biolabs, CA, United States) was used.

### Immunofluorescence

The NPCs were seeded on grass coverslips, and fixed with 4% paraformaldehyde in phosphate buffer saline (PBS) buffer for 20 min and washed with PBS buffer. Then the coverslips were treated by 1% Triton X-100 in PBS buffer for 10 min and washed with PBS buffer. The NPCs were incubated with anti-COL2A1l (sc-52658, Santa Cruz) at 4°C overnight and washed 4 times with PBS buffer. Then second antibodies were used to incubate with cells for 2 h and washed with PBS buffer. The NPCs were stained with DAPI for 10 min and mounted with slide mounting medium. All the antibodies were diluted with 5% Bovine Serum Albumin (Sangon Biotech, Shanghai, China). The Colibriepifluorescence microscope (Carl Zeiss, Jena, Germany) was used to observe the slides and the pictures were analyzed with ImageJ software.

### SiRNAs, Plasmids and Transfection

The siRNAs of kinases and control siRNAs were purchased from RiboBio (Guangzhou, China) and shRNA plasmids were constructed with PLKO.1. SiRNAs were transfected into NPCs with RNAiMAX (Invitrogen) and Opti-MEM medium (Invitrogen). The sequence for the specific siRNAs were as follows: siPLK1, GAU​CAC​CCU​CCU​UAA​AUA​U, sip53, GCA​UGA​ACC​GCC​GGC​CCA​UTT. The mixture of 5 pmol siRNA, 3 µl RNAiMAX and 100 µl Opti-MEM medium was incubated for 5 min and transfected into the cells. 0.4 µg Human PLK1 shRNAs, 0.3 µg packaging plasmid psPAX2 and 0.1 µg lentivirus expression plasmid pMD2G mixed with 5 µl polyethylenimine and 100 µl Opti-MEM medium for 5 min and were transfected into 293T cells to obtain lentiviruses. The sequence for the specific shRNAs were as follows: shPLK1-1, GGC​AAC​CAA​AGT​CGA​ATA​TGA, shPLK1-2, GCA​CCG​CAA​TCA​GGT​CAT​TCA. After ultracentrifuged, the lentiviruses infected NPCs twice and NPCs were screened with puromycin dihydrochloride (Beyotime, Shanghai, China). The surviving cells were cultured as stable mass transfectants.

### Apoptosis Analysis

In order to study the effect of PLK1 kinase on NPCs, we used Annexin V-FITC/propidium iodide (PI) Apoptosis Kit (Multi Science, Hangzhou, China) to detect the treated cells. After collecting the NPCs, the cells were washed with cold PBS twice and added 500 μl Apoptosis Positive Control Solution to resuspend on the ice for 30 min. Then the samples were washed again and added Binding Buffer, 5 μl Annexin V-FITC or 10 μl PI. 5 min later, the samples were detected by a Flow cytometer (BD FACSCANTO II, BD Biosciences, San Jose, CA, United States) and the data were analyzed with the FlowJo software.

### Immunohistochemistry and Staining

After the rats were sacrificed, the tail vertebra tissues were taken out and fixed with 4% paraformaldehyde for 24 h and then decalcified with 12.5% EDTA for about 4 weeks in the decalcification machine. Treat the specimens with different concentrations of ethanol, embed them in paraffin, and cut the specimens into slices with a thickness of 3 μm. These sections were subjected to different staining and immunohistochemistry. The primary antibodies include anti-Aggrecan (13880-1-ap, Proteintech), anti-MMP3 (ab52915, Abcam) and anti-SOX9 (ab185966, Abcam).

### A Rat Model of IVDD

30 male Sprague Dawley rats aged 12–14 weeks were purchased from Shanghai SLAC Laboratory Animal, Co., Ltd. (Shanghai, China), weighing about 320–340 g. After disinfecting the tails of 24 rats, an 18G needle was used to puncture the joints and penetrate into the skin about 5 mm to destroy the NP tissue. The tip of the needle is rotated 360°, placed and held for about 30 s. The remaining six rats served as a control group. 8 weeks later, 24 rats were divided into four groups and injected with Volasertib (15 mg/kg), Pifithrin-α (4 mg/kg) or normal saline through the tail vein, twice a week. After 4 weeks, the rats were painlessly sacrificed and the tail vertebra tissues were taken out and imaged with X-ray and MRI.

### Statistical Analysis

Statistical significant differences were calculated using a Student’s t-test. We use SPSS 19.0 (SPSS, Chicago, IL, United States) to analyses the data. When *p* value was equal to or less than 0.05, it was considered to be statistical significant.

## Data Availability

The original contributions presented in the study are included in the article/Supplementary Material, further inquiries can be directed to the corresponding authors.

## Abbreviations

IVD, intervertebral disc; IVDD, intervertebral disc degeneration; NPC, nucleus pulposus cells; LBP, low back pain; MMP, metalloproteinase; PG, proteoglycan; ECM, extracellular matrix.
