# Supplementary material for: PLK1 Mitigates Intervertebral Disc Degeneration by Delaying Senescence of Nucleus Pulposus Cells
Source: Front Cell Dev Biol. 2022 Mar 14;10:819262. doi: 10.3389/fcell.2022.819262 (PMC8964438; doi:10.3389/fcell.2022.819262)
Supplement: Supplementary file 1 [file DataSheet1.PDF]

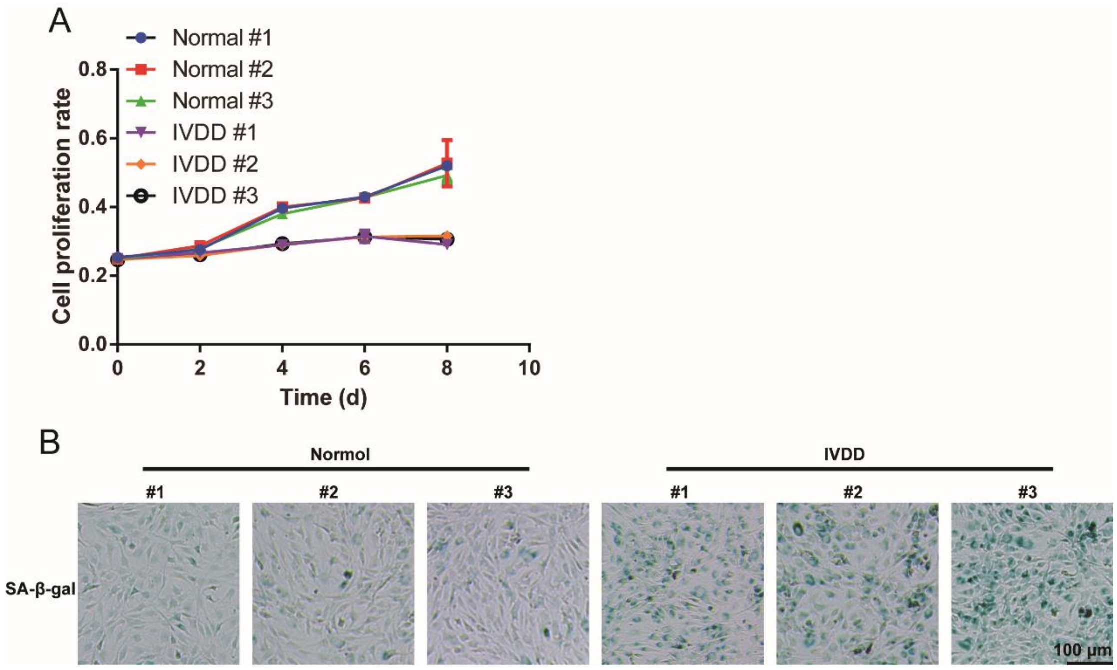

Figure S1. The growth rates and senescence in normal and degenerative NP cells.

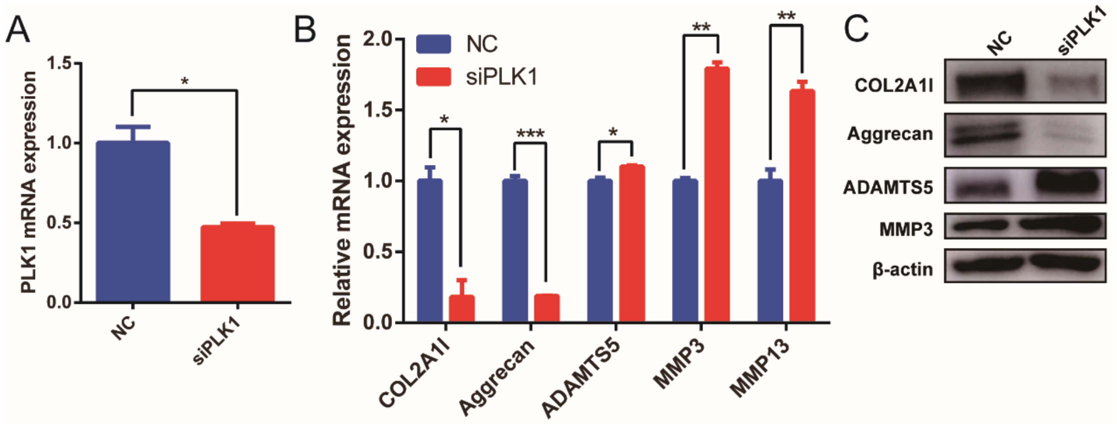

Figure S2. (A) The efficiency of siPLK1 in NPCs. (B and C) The mRNA and protein expression of collagen II, aggrecan, ADAMTS5, MMP3 and MMP13 after PLK1 knockdown.

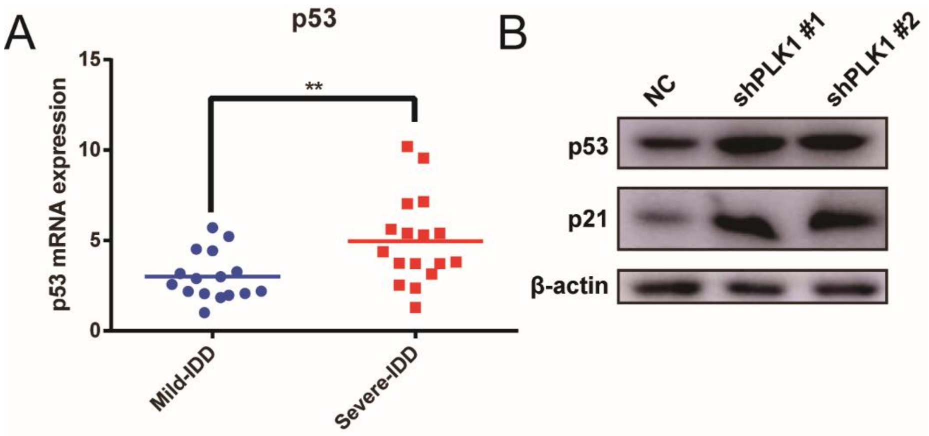

Figure S3. (A) The expression of p53 in the degenerated NPCs. (B) The p53 and p21 protein expression after PLK1 knockdown.

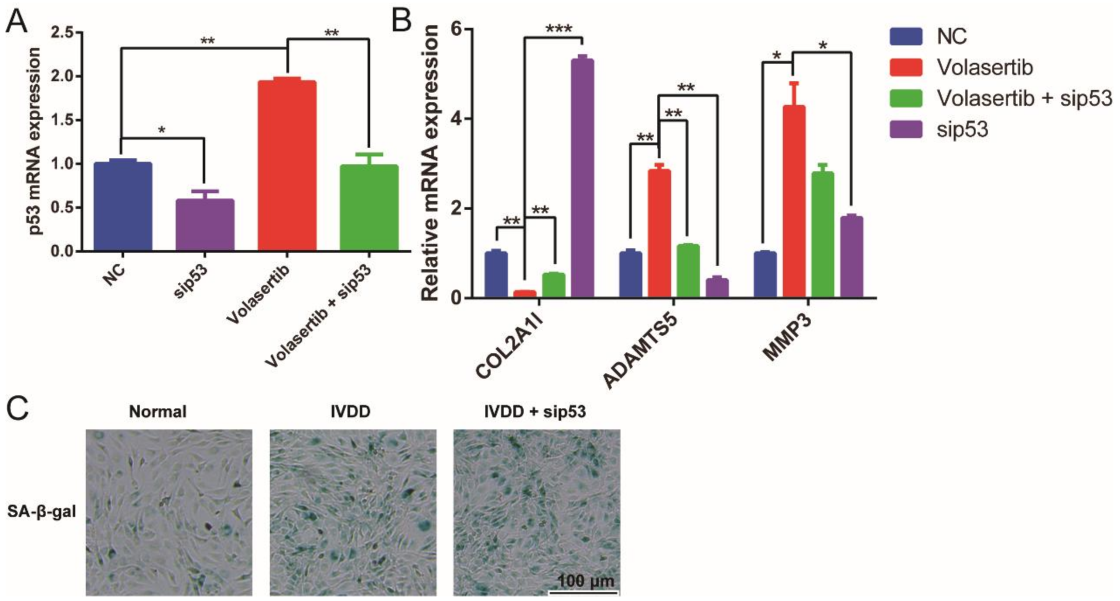

Figure S4. (A) The efficiency of sip53 in NPCs. (B) The mRNA expression of collagen II, ADAMTS5, MMP3 after p53 knockdown. (C) Senescence of NPCs was measured by SA-β-Gal assay after treatment with sip53.
